# Case report: Indocyanine green fluorescence-guided imaging in laparoscope, a more sensitive detection technique of lateral lymph nodes metastases from rectal neuroendocrine tumors

**DOI:** 10.3389/fonc.2022.1101990

**Published:** 2022-12-16

**Authors:** Yueyang Zhang, Yi Zhang, Yi Yang, Zheng Xu, Changyuan Gao, Meixi Liu, Wenjia Zhu, Hong Zhao, Haitao Zhou

**Affiliations:** ^1^ Department of Colorectal Surgery, National Cancer Center, Cancer Hospital, Chinese Academy of Medical Sciences and Peking Union Medical College, Beijing, China; ^2^ Department of Pathology, National Cancer Center, Cancer Hospital, Chinese Academy of Medical Sciences and Peking Union Medical College, Beijing, China; ^3^ Department of Hepatobiliary Surgery, National Cancer Center, Cancer Hospital, Chinese Academy of Medical Sciences and Peking Union Medical College, Beijing, China; ^4^ Department of Nuclear Medicine, Beijing Key Laboratory of Molecular Targeted Diagnosis and Therapy in Nuclear Medicine, Peking Union Medical College Hospital, Chinese Academy of Medical Sciences and Peking Union Medical College, Beijing, China

**Keywords:** rectal neuroendocrine tumors, lateral lymph nodes metastases, lymph nodes dissection, indocyanine green, laparoscope

## Abstract

**Background:**

The diagnosis and surgical strategy of lateral lymph node metastases of rectal neuroendocrine tumors are still controversial. At present, the major diagnostic means rely on imaging examinations, but will be affected by the size of lymph nodes leading to false negativity. We provide a new technique to determine lateral lymph node metastases during surgery.

**Clinical case:**

A 68-year-old man developed abdominal pain, bloating and fever for a month. Colonoscopy revealed the mass is 2.4 cm x 2.0 cm in size, with a wide stratum, poor mobility, and a rough but intact surface mucosa. Therefore, rectal neuroendocrine tumors (R-NET) were diagnosed. Multiple imaging methods, such as CT, octreotide imaging and endoscopic ultrasonography, have not found lateral lymph node metastases from rectal neuroendocrine tumors. But indocyanine green (ICG)-enhanced near-infrared fluorescence-guided imaging during surgery found left lateral lymph nodes metastases, which was proved by postoperative pathological examination.

**Conclusions:**

We believe that applying ICG-enhanced near-infrared fluorescence-guided imaging in laparoscope can improve the detection of positive LLNs in those R-NET patients who did not reveal LNM on imaging examinations.

## Introduction

Lateral lymph nodes (LLNs) metastases are directly correlated with poor prognosis in rectal neuroendocrine tumors (R-NET) (1). Many scholars have begun to pay attention to lateral lymph nodes dissection (LLND) (2, 3). However, there is still no uniform guideline for LLND in R-NET. LLND is recommended for LLNs metastases with clear radiographic evidence which is based on the size of the metastatic lymph nodes (LNs) (2, 4, 5). Nevertheless, Kim B.C. et al. reported that LNs size is not a reliable criterion for predicting lymph node metastases (LNM) in R-NET (6).

Here, we applied a new method of assessing LNM and found a case of R-NET patient with multiple negative imaging examinations (computed tomography (CT), endoscopic ultrasound (EUS), and ^99^Tc^m^-octreotide SPECT developed LLNs metastases. Namely, LLNs metastases are confirmed by applying indocyanine green (ICG)-enhanced near-infrared fluorescence-guided imaging in laparoscope.

## Case description

**Figure 1:**
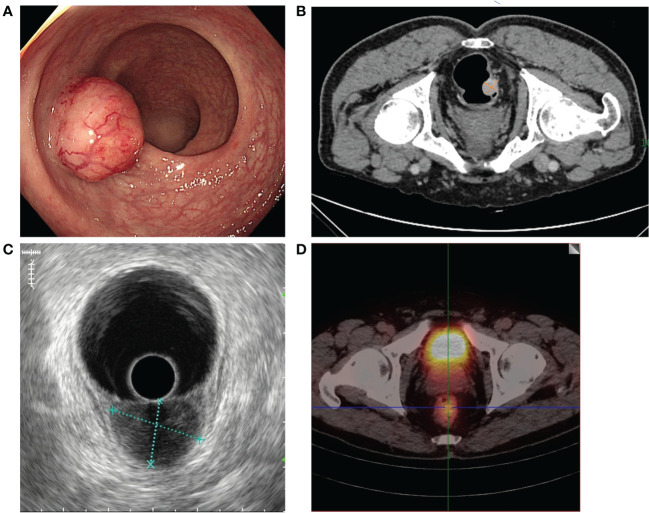
Preoperative endoscopic and imaging examination results. **(A)** Colonoscopy revealed the tumor was 2.4 cm × 2.0 cm in diameter in the lower rectum with a wide stratum. **(B)** CT revealed no LLNs enlargement in the mesorectum and on both sides of the lateral pelvic space. **(C)** The EUS reveals the lesion invades the submucosal layer and no LLNM. **(D)** ^99^Tc^m^-octreotide SPECT revealed high uptake in the rectal cavity and no uptake in either side of the lateral pelvic space.

A 68-year-old man developed abdominal pain, bloating and fever for a month. The rectal touch found a mass (20mm in diameter) in the lower rectum that was located 5cm from the anal verge at the posterior side of the rectum wall. Colonoscopy revealed the mass is 2.4 cm × 2.0 cm in size, with a wide stratum, poor mobility, and a rough but intact surface mucosa (**Figure 1A**). And the biopsy was performed. The pathological diagnosis of NET was confirmed. Considering that the maximum diameter of the tumor is 2.4 cm, further radical surgical treatment is necessary for this patient. Low anterior resection (LAR) and regional LNs dissection is the recommended surgical method. More detailed imaging examinations were therefore performed to evaluate the condition of LLNs metastases. A CT examination revealed the tumor is significantly enhanced and the fat gap around the tumor is blurred (**Figure 1B**). The endoscopic ultrasound (EUS) reveals the lesion invades the submucosal layer, and some levels are suspected of invading the innate muscular layer (**Figure 1C**). But, neither CT nor EUS reveals pelvic, abdominal, or retroperitoneal enlarged LNs. ^99^Tc^m^-octreotide SPECT images revealed high uptake in the rectal cavity which is consistent with neuroendocrine tumor manifestations (**Figure 1D**). But there are no high-uptake foci in the regional LNs. Therefore, multiple imaging examinations cannot determine the LLNs metastases of the patient.

**Figure 2:**
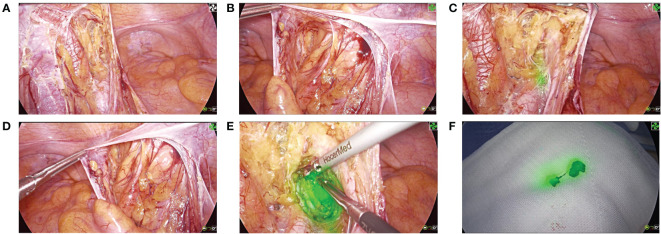
4K fluorescence laparoscopic surgical procedure. **(A)** Expose the region of the left iliac vessel. **(B)** Expose the region of the right iliac vessel. **(C)** Positive ICG-fluorescent staining of the left LLNs. **(D)** Negative fluorescent staining of the right LLNs. **(E)** Dissect the left LLNs. **(F)** Specimen of the left LLNs.

Two weeks later, 4K fluorescent laparoscopic radical resection of rectal cancer was performed. Before total mesorectal excision (TME), anoscopic injection of ICG 1ml(2.5mg) at 0.5cm above, below, on the left, and on the right side of the tumor, respectively. After opening the peritoneum, the internal iliac vessels were subsequently cleared from the lymphatic tissue at a safe distance from the lateral side of the pelvic plexus (**Figures 2A, B**). In the obturator space, fluorescence staining of the left LLNs is visible (**Figure 2C**). In contrast, LLNs on the right side are not visible with fluorescence staining (**Figure 2D**). Therefore, the left LLND was performed (**Figures 2E, F**). Following completion of the left LLND, only external vessels, internal iliac vessels and their branches, the obturator nerves, and the pelvic plexus remained. The procedure after lymphadenectomy is conventional AR and regional LNs dissection. The operative time was 207min, and the intraoperative blood loss was 100ml. The patient recovered well from surgery and was discharged on postoperative day 9. The adjuvant therapy was not performed.

**Figure 3:**
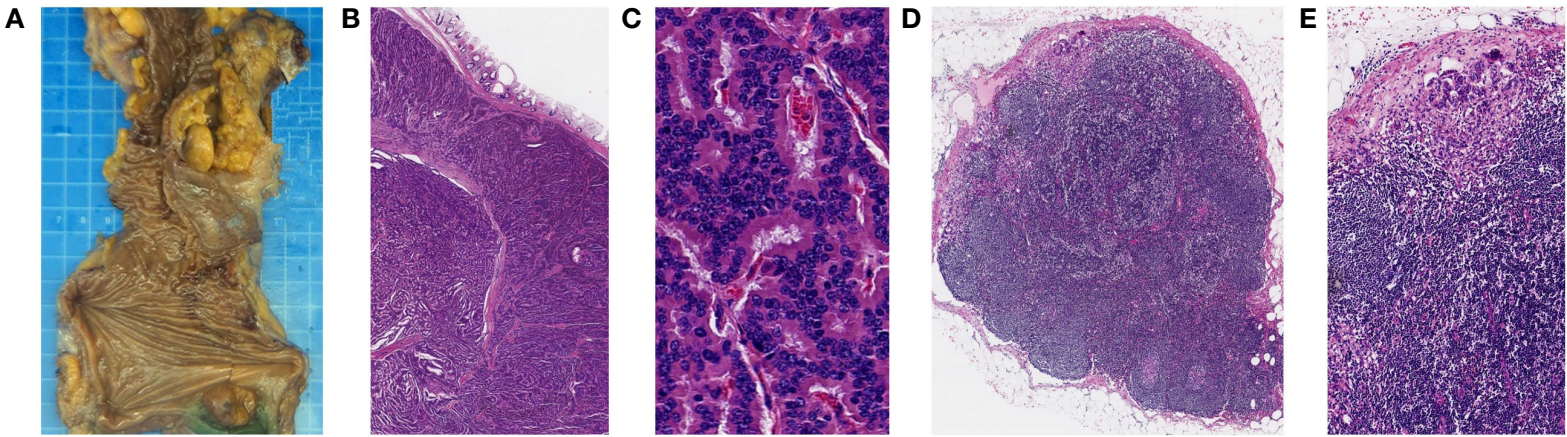
Histopathological findings of the resected specimen (primary tumor and left LLNs). **(A)** Macroscopic findings of the resected specimen. **(B, C)** HE staining showed that the primary tumor cells spread in the organ-like features, trabecular, glandular, and gyri-like (B magnification, x20, C magnification, x400). **(D, E)** HE staining showed that left LLNs have similar findings to those of the primary tumor (**D** magnification, x20, **E** magnification, x100).

**Figure 4:**
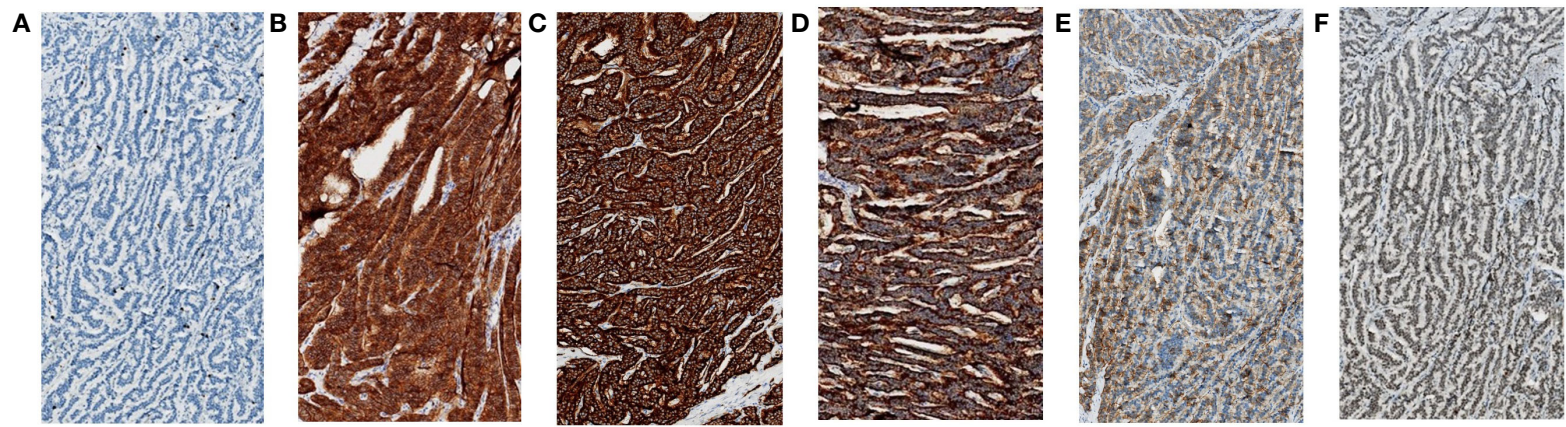
Immunohistochemical staining (magnification, x100) of the resected specimen (primary tumor and left LLNs). **(A)** The Ki-67 index was 1-2%. **(B)** The CD56 was 3+. **(C)** The synaptophysin was 3+. **(D)** The CgA was 3+. **(E)** The SSTR2 was 2+. **(F)** The Rb-1 was 2+.

The resected specimen indicated that the primary tumor was 20mm in diameter and invaded peri-intestinal adipose tissue (**Figure 3A**). Hematoxylin and eosin (HE) staining of the primary tumor and LLNs showed that the tumor cells spread in the organ-like features, trabecular, glandular, gyri-like (**Figures 3B-E**). The vessel carcinoma embolus and nerve invasion were detected. A pathological diagnosis of NET G1(pT3N1) was confirmed according to a Ki-67 index of 1-2% (**Figure 4A**). The macroscopic and microscopic findings of the specimen revealed that the surgical margin was negative. One of the 6 LNs in the mesorectum contained metastases from the NET, and one of the 6 LLNs on the left side contained metastases from the NET. More results of immunohistochemistry are shown in **Figure 4** and **Supplementary Table 1**. The patient was followed up with abdominal CT every 3 months. Three months after the operation, he showed symptoms of chronic diarrhea, but there was no fecal incontinence, and his voiding and sexual functions were also preserved. At the six-month follow-up, the symptoms of diarrhea were slightly relieved. And no local recurrence or distant metastases had been found. More details about the timeline with relevant data are shown in **Supplementary Table 2**.

## Discussion

The incidence of gastroenteropancreatic (GEP)-NETs has increased significantly in the past 20 years (7–9), which is related to the abundance of examination methods and the improvement of tumor screening. Among all GEP-NET patients, the incidence of R-NET has shown the second highest increase in recent years according to the major data emerging from both the US National Cancer Institute Surveillance, Epidemiology and End Results database (SEER) and national cancer registries in Western Europe (7, 8). In the National Comprehensive Cancer Network guidelines, R-NETs >2 cm with invasion into the muscularis propria or LNM should be treated with LAR (10). In the European Neuroendocrine Tumor Society guidelines and the North American Neuroendocrine Tumor Society consensus guidelines, patients with R-NETs >2 cm and 1-2 cm NETs with muscular invasion or positive lymph nodes (LNs) are recommended to undergo radical resection with LN dissection (11, 12). The incidences of LNM in tumors of different sizes are 1.0% (≤5 mm), 8.4% (6-10 mm), 54.5% (11-20 mm) and 66.7% (≥21 mm) (13). LNs dissection refers to regional LNs dissection of the rectum. However, the definition of regional LNs is currently controversial (14, 15). In the West, scholars deem that LLND has complications such as long operation time, increased intraoperative bleeding, postoperative impact on urination and sexual function, and cannot improve the survival rate of patients (16, 17). Therefore, the standard treatment used usually includes neoadjuvant chemoradiotherapy (NCRT) and total mesorectal excision (TME) (15, 18). The addition of NCRT has decreased the incidence of local recurrence from 11.3% to 5.8% (19). In contrast, Japanese scholars generally believe that the LLNs are regional LNs of rectal cancer (4), so preventive LLND is performed for rectal cancer below peritoneal reflection (20).

The concept of LLNs was elaborated by Villemin and Montagn’e in 1925 (21). They demonstrated that the low rectum was obvious in its lateral lymphatic drainage to the iliac nodes. Meanwhile, in Japan, Senba Y. described injecting dye into fetal cadavers to demonstrate that these lymphatic vessels are distributed in the internal iliac and obturator spaces (22). Although they vary greatly, these pathways have been confirmed by recent lymphatic scintigraphy techniques (23). Conventional TME surgery does not remove the LLNs, which may be one of the reasons for local recurrence after the surgery. Akiyoshi, Ueno et al. performed LLND in patients with suspected LLNs metastases based on CT or magnetic resonance imaging (MRI) before chemoradiotherapy (CRT) and total rectal resection (TME) in patients without suspected LLNs metastases (5). They found no recurrence in the former, while three recurrences occurred in the latter. This indicates LPLD might improve regional control and survival of patients with LLNs metastases in advanced low rectal cancer treated with preoperative CRT. But there is no clear expert consensus or guidelines regarding the diagnosis and treatment of R-NETs with simultaneous LLNs metastases. Meanwhile R-NETs are relatively inert and low malignancy. So whether LLND could improve the prognosis is controversial.

From previous reports, 5.8–6.5% of patients with R-NETs were confirmed to have LLNs metastases after rectal resection (24, 25). 32 reported cases of LLNs metastases from R-NETs have been reported (3, 24–27). Among them, Liu, X., et al. reported 3 cases of R-NET of LLNs metastases. They proposed that transanal local resection (TLR) combined with LLND is worth applying to patients with R-NET who is solely suspected LLNs metastases and without mesorectal LNM. At the same time, we retrospectively analyzed the LNM of R -NET patients in the National Cancer Center, Chinese Academy of Medical Sciences, Beijing, from January 2000 to December 2018. Of the 113 patients with R-NET, whose mean tumor size was 2.29 cm, 12 developed LLNs metastases. The metastatic rate was 10.6%, which was indeed higher than the 5.8% - 6.5% reported in the literature. The evaluation method for lateral lymph node metastases is to dissect the LLNs and perform pathological examination if the suspected lateral lymph node metastases is found on preoperative imaging examinations. If the pathological examination is positive, lateral lymph node metastases will be confirmed. Considering that 54 patients underwent endoscopic local resection (EMR, ESD) and 15 patients did not undergo surgery due to distant metastases, we deem that many patients with LLNs metastases have been missed. The reason may be that the preoperative LNs evaluation of R-NET relies primarily on imaging diagnosis.

CT, especially images using a scan width of 5mm, is generally applied as a routine approach to determine operative indications or preoperative staging. However, it has been reported that CT is difficult to predict LNM in R-NET patients (24). By contrast, MRI is well known to provide higher-quality images to evaluate the circumferential margin and detect the existence of positive LNs than CT. However, due to economic considerations, MRI is usually not performed in patients with R-NET who have negative LNs on CT examination. And Liu, X., et al. mentioned CT and MRI are less sensitive to the diagnosis of LNM, especially LLNs metastases (3). This may be due to CT and MR relying heavily on the size of the LNs to determine whether there are LNM. Kim, B.C., et al. indicated the size of LNs containing metastases is highly variable, with some being very small (6). Therefore, LNs size alone is not an adequate predictor of tumor metastases in R-NET. Another imaging examination, 68Ga-DOTANOC PET-CT, is a promising tool for detecting LNM in R-NETs with high sensitivity and specificity in visual assessment (28). However, previous studies have reported CT and MRI as superior to octreotide scan for detecting metastases (29, 30).

In summary, the recommended imaging examinations have their shortcomings. In our case, we reported a patient with R-NET who did not reveal LNM on either recommended imaging examinations or one endoscopic examination. To the best of our knowledge, there are no detailed reports in English about the similar situation. Meanwhile, we provide a new method to help surgeons accurately determine LLNs metastases during surgery, and further perform LLND to improve the prognosis of patients. That is, ICG fluorescence imaging is used to confirm the lymphatic drainage of tumor tissue, and then to determine suspicious LNs. ICG has been approved by the FDA for clinical use in humans in 1959 and since then it has been applied for a broad range of surgical indications (31). It is a water-soluble fluorescent tracer. After injection into the blood, most (98%) were bounded to albumin or other carriers until bile excretion (32). The bounded ICG remains in the blood vessels and allows real-time fluorescent imaging of vasculature and lymphatic structures within 1 minute. This approach has been applied to advanced middle-low rectal cancer. Zhou, S.C., et al. reported that compared to the non-ICG group, the ICG group had a significantly larger number of LLNs harvested (33). This approach has multiple advantages: First of all, it complements the lack of LLNs metastases solely detected by imaging by providing a more informative map of the lymphatic structures. Secondly, it allows us to preserve more negative LNs. Ultimately, this method is economical and easy to perform by the surgeon intraoperatively. The important role of LNs in tumor immunotherapy has been reported (34, 35). Therefore, our method can accurately resect positive LLNs and retain those negative LNs with potential immune function.

Therefore, based on the existing literature and a review of the clinical data of R-NET in our center, we believe that applying ICG-enhanced near-infrared fluorescence-guided imaging in laparoscope can improve the detection of positive LLNs in those R-NET patients who did not reveal LNM on imaging examinations. Certainly, we still need more cases and retrospective studies to improve evidence-based medicine and guide clinical work more clearly.

## Data availability statement

The original contributions presented in the study are included in the article/**Supplementary Material**. further inquiries can be directed to the corresponding authors.

## Ethics statement

The study involving human participants was reviewed and approved by Cancer Hospital, Chinese Academy of Medical Sciences. Written informed consent requirements were waived due to the retrospective character of this study.

## Author contributions

ZHT and ZH contributed to conception and design of the study. ZYY, XZ and GCY collected the clinical information of this case report. ZYY wrote the first draft of the manuscript. ZY wrote the pathological section of the manuscript. YY wrote the radiological section of the manuscript. ZYY, ZY and YY contributed equally to this work and share first authorship. ZHT and ZH contributed to manuscript revision, read, and approved the submitted version. All authors contributed to the article and approved the submitted version.
