# Supplementary material for: Case report: Indocyanine green fluorescence-guided imaging in laparoscope, a more sensitive detection technique of lateral lymph nodes metastases from rectal neuroendocrine tumors
Source: Front Oncol. 2022 Dec 16;12:1101990. doi: 10.3389/fonc.2022.1101990 (PMC9800599; doi:10.3389/fonc.2022.1101990)
Supplement: Supplementary file 2 [file Table_2.docx]

| Time | Examination or Treatment | Results |
| --- | --- | --- |
| 2022.04.19 | Colonoscopy | The mass is 2.4 cm $\times$ 2.0 cm in size, with a wide stratum, poor mobility, and a rough but intact surface mucosa; |
| 2022.04.19 | EUS | The lesion invades the submucosal layer, and some levels are suspected of invading the innate muscular layer. |
| 2022.04.20 | Pathological Examination | Rectal NET was confirmed. |
| 2022.04.25 | 99Tcm-octreotide SPECT | High uptake in the rectal cavity which is consistent with NET. |
| 2022.06.08 | Admission | |
| 2022.06.14 | Indocyanine green fluorescence-guided surgery | Radical resection of rectal NET and left lateral lymph node dissection. |
| 2022.06.15 | Postoperative day 1 | The total volume of intraperitoneal drainage was 420ml.  No fever. No postoperative defecation and exhaustion. |
| 2022.06.17 | Postoperative day 3 | The total volume of intraperitoneal drainage was 120ml.  Postoperative defecation and exhaustion. |
| 2022.06.20 | Postoperative day 6 | Oral intake was started. |
| 2022.06.21 | Pathological Examination | Positive left lateral lymph node was confirmed |
| 2022.06.23 | Being discharged | |

Supplementary Table 2. A timeline table showing relevant data.
